# Chronic cough in postmenopausal women and its associations to climacteric symptoms

**DOI:** 10.1186/s12905-023-02225-2

**Published:** 2023-03-08

**Authors:** Volker Ziller, Thea Sophie Oppermann, Werner Cassel, Olaf Hildebrandt, Rolf F. Kroidl, Ulrich Koehler

**Affiliations:** 1 Clinic for Gynecology and Obstetrics, Department of Endocrinology, Reproductive Medicine and Osteology, University Hospital Gießen and Marburg, Philipps-University of Marburg, Baldingerstrasse 1, 35043 Marburg, Germany; 2 University Hospital Gießen and Marburg, 35043 Marburg, Germany; 3 Clinic for Internal Medicine, SP Pneumology, Intensive Care and Sleep Medicine, University Hospital Gießen and Marburg, Philipps-University of Marburg, 35043 Marburg, Germany; 4 Lung Center Stade, Frommholdstrasse 71, 21680 Stade, Germany

**Keywords:** Chronic cough, Menopause, Menopause Rating Scale (MRS), Leicester Cough Questionnaire (LCQ)

## Abstract

**Background:**

Postmenopausal women often have chronic cough. Hormonal changes might be affecting lung function and the mucous membrane of the airways, causing hypersensitivity of the cough reflex. Therefore, postmenopausal hormonal changes could play a key role in the association between increased cough and menopause. The aim of this study is to evaluate the relation of chronic cough and postmenopausal symptoms.

**Methods:**

We performed a questionnaire-based cohort study in generally healthy postmenopausal women (age 45–65 years). Women with cough explained by a pre-existing diagnosis were excluded. Comorbidities, medication and baseline data were collected. The Menopause Rating Scale II (MRS II) was combined with the Leicester Cough Questionnaire. Groups were divided in chronic cough versus non-coughing participants, chronic cough was defined as symptoms over 8 weeks. We performed correlations and logistic regression for predicting cough based on postmenopausal symptoms.

**Results:**

Sixty-six of 200 women (33%) reported symptoms of chronic cough over 8 weeks. No significant differences in baseline data (age, BMI, onset of menopause, years since menopause, concomitant diseases, and medication) were found between coughing and non-coughing women. The MRS II showed higher menopausal symptoms in patients with cough, with significant differences in 2 of the 3 MRS-domains (urogenital (*p* < 0.001) and somato-vegetative (*p* < 0.001)). Climacteric symptoms correlated strongly with parameters of cough (*p* < 0.001). On the basis of the MRS total score (*p* < 0.001) and the somato-vegetative and urogenital domains (*p* < 0.05), the prediction for respiratory complaints could be shown.

**Discussion:**

Chronic cough was significantly associated with menopausal symptoms. Therefore chronic cough as a possible climacteric symptom and its underlying mechanisms should be further explored.

## Background

Chronic cough in adults is defined by its persistence for at least 8 weeks [1–4]. Chronic cough is associated with a significant impairment of physical performance and quality of life [5–11]. In a relevant number of patients, etiology of chronic cough remains unclear despite extensive diagnostics. The cough receptors’ hypersensitivity seem to be an underlying factor. Middle-aged women who complain of chronic cough are suspected to have a causal relationship between coughing irritation and post-menopausal changes [2, 12–15].

Menopause is a complex phenomenon. The cessation of the ovarian activity and the subsequent lack of sex steroids, especially estrogen and progesterone, results in substantial changes in the female body that are generally summarized as "climacteric syndrome." Typically, among other symptoms, this includes hot flushes, sweats and vaginal atrophy, as well as muscle and joint complaints [16–19]. The changes in skin, connective tissue and mucous membranes from the lack of estrogen could also manifest in the respiratory tract. As estrogen deficiency leads to atrophy of vaginal epithelia, reduction or modification in mucus, ciliae or changes in sensitivity in respiratory tissue could cause a reduction in lung function and lead to chronic cough [20–26]. If postmenopausal estrogen deficiency plays a part in the development of cough the extend of climacteric symptoms should be correlated to symptoms of cough.

It was therefore the aim of this study to investigate the relationship between postmenopausal symptoms and chronic cough.

## Methods

We performed a questionnaire-based cohort study in postmenopausal women recruited within the outpatient consultation at the University Hospital Marburg, Germany. The women visited the gynecological department for numerous medical reasons, but the majority took part in routine checkups or menopausal counseling including bone densitometry (in our hospital performed by the department of gynecological endocrinology). Participants between the ages of 45 and 65 were offered participation, although the last menstruation had to be at least one year ago.

We excluded women with irregular bleeding, smokers, history of any recent sex steroid treatment, including Menopause Hormone Therapy (MHT) or any differential diagnosis that might be connected to chronic cough (e.g. cancer, chronic bronchitis, gastro-esophageal reflux, chronic heart failure, therapy with ace-inhibitors).

### Study procedures

After written informed consent, specified inclusion and exclusion criteria, as well as the differential diagnoses for chronic cough, were queried and documented. When all criteria for participation were met, the severity of menopausal symptoms and the severity of cough were measured using the Menopause Rating Scale II and the Leicester Cough Questionnaire LCQ [27, 28].

### Definition of chronic cough

Chronic cough was defined as any cough that persisted for at least eight weeks [3, 29, 30]. The subjects had to give a time indication of the duration of their cough, if it was present.

If this duration was reported over 8 weeks or longer, we classified them as coughing subjects. There was no minimum level of symptoms, because minor complaints in the context of coughing should also be recorded.

### Menopause Rating Scale II (MRS II)

The MRS II is a simple, flexible, and quickly implementable tool for evaluating postmenopausal symptoms [28, 31]. The 11 symptoms are given on a 5-point Likert scale (0 = no complaints to 4 = very severe complaints) [32]. The total score can range from 0 (asymptomatic) to 44 (highest level of complaints). A low scale value correlates with a higher quality of life.^31^ Within the MRS II, 3 subgroups can be distinguished. The “somato-vegetative” subgroup suffers increasingly from sleep disorders, heart problems, hot flushes and joint/muscle problems (items 1, 2, 3, and 11 with 0–16 possible points). The “urogenital” type focuses on complaints of the urinary tract, vagina and sexuality (items 8, 9, and 10 with 0–12 possible points). The “mental” subgroup include exhaustion, irritability, anxiety and mood (items 4, 5, 6, and 7 with 0–16 possible points) [31]. To calculate the total score for the 3 subscales, the degrees of severity of the associated items are added. To calculate the total number of points, subscale values are combined.

### Leicester Cough Questionnaire (LCQ)

The LCQ consists of 19 items that cover a physical (8 items), mental (7 items), and social (4 items) domains. The physical condition of the patient is inquired through items 1, 2, 3, 9, 10, 11, 14, and 15 and refers to symptoms that can be associated with cough, including abdominal/chest pain, the production of sputum, fatigue, sleep disorders, hoarseness and changed performance. In addition, certain situations that trigger the cough are recorded. Items 4, 5, 6, 12, 13, 16, and 17 deal with mental aspects: the ability to control the cough reflex and the emotions associated with the symptoms (fears, embarrassment, discouragement, frustration, and worry) play a role in the question selection. Social effects are covered by questions 7, 8, 18, and 19. In this case, the influence of cough symptoms on everyday situations, relationships with family members and on enjoyment of life is asked [27].

The 3 domains are evenly distributed across the entire questionnaire. Scores are calculated as a mean of each domain and the total score is calculated by adding every domain score [27].

### Statistics

To distinguish a correlation of r ≥ 0.2 with sufficient test strength (0.8) from r = 0, an inclusion of n = 200 patients was planned. The statistical evaluation was carried out with IBM SPSS Statistics Version 22 (IBM GmbH). Due to the high number of cases of n = 200, parametric test methods were used for descriptive and inferential statistical analyzes. All variables were checked for normal distribution, skewness, and kurtosis. If there was a significant deviation from a normal distribution with very high skew values (> 2), then after visually inspecting the distribution, these parameters were either reverted to non-parametric evaluation methods or the variables were transformed, thus making an approximate normal distribution for the transformed variables. After defining two cohorts (cough versus non-cough) groups were analyzed by comparisons of means. Correlation analysis and logistic regression were applied to the whole cohort.

### Ethics

The study was conducted in accordance to the guidelines and with approval of the local ethics committee of the Philipps-University of Marburg.

## Results

There were 205 subjects interviewed and included after positive prescreening between July 6th, 2017 and October 18th, 2019. Five test subjects had to be excluded due to differential diagnoses afterwards. Finally, 200 women were included in the analysis.

Of the 200, 66 (33%) patients had symptoms of chronic cough over the last eight weeks. They were compared to the 134 (67%) that had none. There was no statistical difference between the two groups concerning their baseline datap (Table 1).

**Table 1:** Baseline characteristics

n = 200	With cough (n = 66)		Without cough (n = 134)			
	Mean values	Standard deviation	Mean values	Standard deviation	t-test, *p*-value	95% CI
Age (years)	57.18	3.56	57.17	3.91	0.99	− 1.12; 1.14
Weight (kg)	70.38	13.83	67.82	12.23	0.19	− 1.08; 1.10
Height (cm)	165.5	6.10	165.74	6.19	0.80	− 2.07; 1.59
Bmi *(*kg/m^2^)	25.63	4.44	24.65	4.03	0.12	− 0.26; 2.21
Years since menopause	8.05	5.60	8.15	6.27	0.91	− 1.90; 1.70
Number of diseases	1.23	0.97	1.01	1.00	0.16	− 0.08; 0.51
Number of medications	1.10	1.45	0.79	0.99	0.09	− 0.05; 0.65

The mean age of the patients in both groups was 57.2 years. They did not differ significantly in terms of weight, height, time since menopause, number of previous illnesses and co-medication. The frequencies of the comorbidities hypothyroidism, osteoporosis, osteopenia and having a hysterectomy did not differ significantly according to the Fisher`s exact test (*p* > 0.05).

The age at the beginning of cough was estimated at 52.6 years on average. The coughing prevailed on average for 4.7 years. In comparison, the subjects entered menopause at 49.14 years on average and the cough started at 52.6 years on average (Table 2).

**Table 2:** Ages and years since onset of menopause and cough symptoms (n = 66)

N = 66	Years since menopause	Years onset of cough	Age onset of menopause (years)	Age onset of cough (years)
Mean value	8.05	4.74	49.14	52.60
Standard deviation	5.60	4.50	4.85	5.17
Minimum	1	0.15	36	36
Maximum	24	20	57	61

### MRS results

Table 3 and 4 list the individual results of the MRS II comparing the 2 cohorts. In in all three domains of the MRS there were statistically significant differences in MRS scores between the subjects with versus without cough. A significant difference in mean MRS value among women in the cough group compared to women in the no-cough group was found in the following symptoms: heart discomfort, sleep problems, physical/mental exhaustion, sexual problems, vaginal dryness and joint/muscle complaints (Table 3).

**Table 3:** comparison of mean values and t-test of single MRS items (n = 66 with cough, n = 134 without cough)

MRS items	Mean values with coughing (n = 66)	Mean values without coughing (n = 134)	Difference	t-test *p*-value
(1) Hot flushes, sweating	1.39	1.19	0.2	0.192
(2) Heart discomfort	0.88	0.51	0.37	0.007
(3) Sleep problems	1.92	1.34	0.58	0.001
(4) Depressive mood	0.85	0.66	0.19	0.164
(5) Irritability	0.86	0.80	0.06	0.640
(6) Anxiety	0.76	0.51	0.25	0.054
(7) Physical/mental exhaustion	1.30	0.91	0.39	0.007
(8) Sexual problems	1.32	0.82	0.5	0.002
(9) Bladder problems	0.70	0.46	0.24	0.053
(10) Vaginal dryness	1.58	1.13	0.45	0.009
(11) Joint/muscular discomfort	1.58	1.17	0.41	0.031

**Table 4:** Mean values and t-test of total score and MRS domains (n = 66 with cough, n = 134 without cough)

MRS	Mean values with cough (n = 66)	Mean values without cough (n = 134)	Difference	t-test *p*-value
MRS total score	13.14	9.51	3.63	< 0.001
MRS somato-veg. (items 1, 2, 3, 11)	5.77	4.22	1.55	< 0.001
MRS urogenital (items 8, 9, 10)	3.59	2.42	1.17	< 0.001
MRS mental (items 4, 5, 6, 7)	3.77	2.87	0.9	0.036

**Fig. 1:**
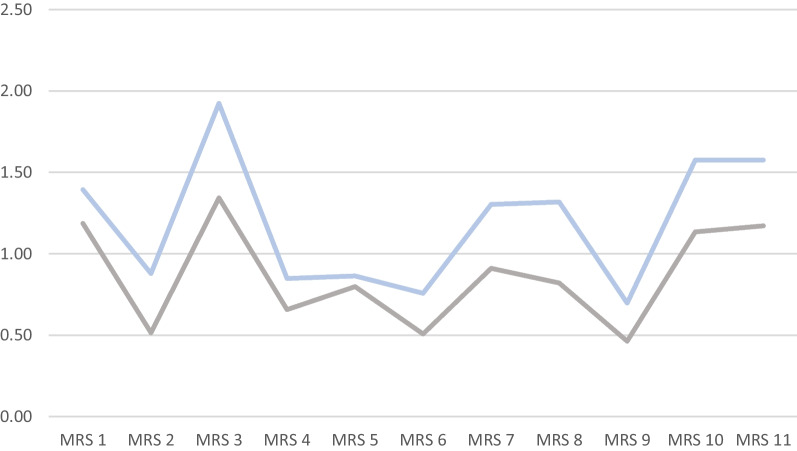
comparison of mean values of single MRS items (n = 66 with cough (blue), n = 134 without cough (gray)) (graphic program: MS Office)

Figure 1 shows the mean value comparison of the individual MRS items in both groups.

### LCQ results

The LCQ score was 17.81 in the subjects with a chronic cough and 20.79 in the subjects without cough (*p* < 0.001). We compared the mean values of the subscale values for the LCQ domains (physically, socially, and mentaly). Comparing the subjects with cough versus no cough resulted in a score of 5.78 versus 6.80 (*p* < 0.001), 5.88 versus 6.99 (*p* < 0.001) and 6.16 versus 7.0 (*p* < 0.001), respectively (Table 5).

**Table 5:** Mean values and t-test of total score and LCQ domains

LCQ	Mean values with coughing (SD) (n = 66)	Mean values without coughing (SD) (n = 134)	t-test *p*-value
LCQ total score	17.81 (2.35)	20.79 (0.24)	< 0.001
LCQ physical	5.78 (0.22)	6.80 (0.79)	< 0.001
LCQ mental	6.16 (0.80)	7.00 (0.00)	< 0.001
LCQ social (items 7, 8, 18, 19)	5.88 (0.93)	6.99 (0.05)	< 0.001

**Fig. 2:**
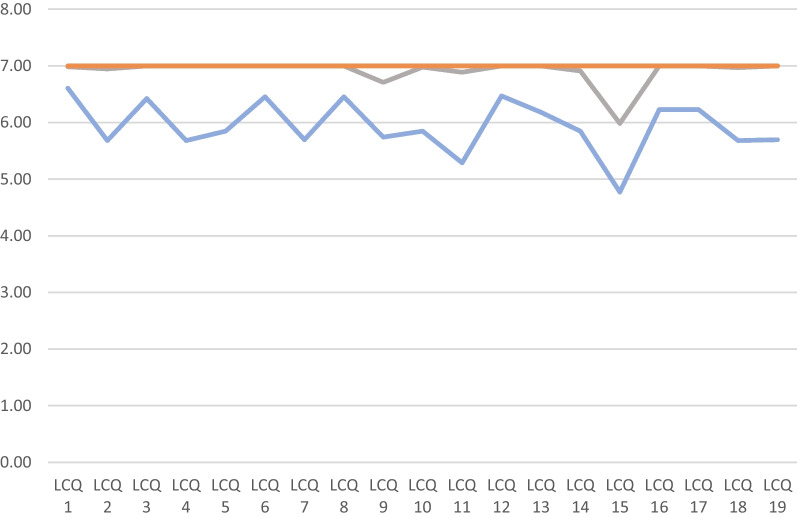
comparison of mean values of single LCQ items. (n = 66 with cough (blue), n = 134 without cough (gray), possible points (orange)) (graphic program: MS Office)

The results of the individual LCQ-items in comparison of the two groups are shown in Fig. 2 and Table 6 (*p* < 0.001).

**Table 6:** Mean values and t-test of single LCQ items

n = 200	Mean values with cough (n = 66)	Mean values without cough (n = 134)	Diff	t-test *p*-value
(1) Chest/abdominal pain	6.61	6.99	0.38	< 0.001
(2) Sputum production	5.68	6.95	1.27	< 0.001
(3) Fatigue	6.42	7.00	0.58	< 0.001
(4) Cough control	5.68	7.00	1.32	< 0.001
(5) Inconvenience	5.85	7.00	1.15	< 0.001
(6) Anxiety	6.45	7.00	0.55	< 0.001
(7) Disturbing factor for oneself	5.70	7.00	1.30	< 0.001
(8) Mood impairment	6.45	7.00	0.55	< 0.001
(9) Trigger paint, fumes	5.74	6.71	0.97	< 0.001
(10) Sleep problems	5.85	6.98	1.13	< 0.001
(11) Coughing fits	5.29	6.89	1.60	< 0.001
(12) Discouragement	6.47	7.00	0.53	< 0.001
(13) Annoyance	6.18	7.00	0.82	< 0.001
(14) Hoarseness	5.86	6.91	1.05	< 0.001
(15) Energy, drive	4.77	5.99	1.22	< 0.001
(16) Worry about illness	6.23	7.00	0.77	< 0.001
(17) Fear of condemnation	6.23	7.00	0.77	< 0.001
(18) Interruption of conversation	5.68	6.97	1.29	< 0.001
(19) Disruptive factor for others	5.70	7.00	1.30	< 0.001

### Correlation MRS and LCQ

A correlation analysis of the MRS scale versus the LCQ scale was applied to the whole cohort. Results are presented in Table 7. There is a negative correlation of -0.428 between the total LCQ and MRS scores. After controlling for age, BMI and years since menopause, the result remains significant (− 0.429). Correlating the overall MRS score with the three LCQ domains results in a negative correlation from − 0.336 to − 0.481. In particular, the LCQ domain for the physical symptoms of cough correlates negatively with the entire postmenopausal symptoms. As expected LCQ total score correlates very high with the mental, physical and social dimension of cough. A high correlation between the MRS total score and the three MRS-D domains could be shown.

**Table 7:** Correlations of the results of MRS and LCQ (n = 200)

N = 200	LCQ total score	MRS total score	LCQ social	LCQ mental	LCQ physical	MRS somato-vegetative	MRS uro-genital	MRS mental
LCQ total score	1							
MRS total score	− 0.428	1						
LCQ social	0.953	− 0.336	1					
LCQ mental	0.959	− 0.367	0.928	1				
LCQ physical	0.955	− 0.481	0.848	0.846	1			
MRS somato-vegetative	− 0.382	0.841	− 0.296	− 0.323	− 0.436	1		
MRS urogenital	− 0.365	0.734	− 0.294	− 0.322	− 0.399	0.431	1	
MRS mental	− 0.299	0.844	− 0.233	− 0.254	− 0.339	0.558	0.449	1

### Logistic regression

A logistic regression to predict cough based on the presence of postmenopausal symptoms was performed. At the level of the individual items, no significant predictive value for chronic cough could be recorded. However, on the basis of the MRS total score and the individual domains, highly significant results with regard to the total score (*p* < 0.001) and significant results with regard to the somatovegetative and urogenital domains (*p* < 0.05) remained (Table 8).

**Table 8:** Coefficient table of the logistic regression to predict cough based on the MRS total score and the MRS domains (n = 200)

n = 200	rc	*p*-value	Odds ratio	95% CI
MRS total score	0.09	< 0.001	1.091	1.040–1.144
MRS somato-vegetative (items 1, 2, 3, 11)	0.15	0.024	1.164	1.020–1.328
MRS urogenital (items 8, 9, 10)	0.18	0.025	1.195	1.023–1.396
MRS mental (items 4, 5, 6, 7)	0.04	0.600	0.965	0.844–1.103

*Rc* Regression coeffiecient; *CI* Confidence interval

## Discussion

Although a variety of diseases can be linked to chronic cough, it is becoming increasingly clear that that the majority of adult patients who have chronic cough as their main complaint present a common clinical picture [4, 33]. They often complain about a pronounced sensitivity when breathing in environmental irritants such as perfume, bleach or cold air that cause a tickling/irritable sensation in the skin, sore throat and coughing. Features that indicated increased sensitivity of neural pathways mediating the cough [34]. In addition, there is a unique epidemiology: two-thirds of patients are female and the highest prevalence in the fifties and sixties [35]. The main finding of this study is a strong association between cough and climacteric symptoms in postmenopausal women. The women in this study were a generally healthy cohort who mostly visited the outpatient department for preventive or prophylactic exams, as well as for menopausal complaints. Of the 200 postmenopausal women, 33%reported cough over at least 8 weeks, regarding LCQ-scores symptoms were rather mild but differed significantly from non-coughing women. When comparing these to ‘women not presenting with chronic cough’, they did not differ in baseline characteristics such as age, weight, number of diseases or years since onset of menopause (Table 1). In the affected women, the cough started an average of 3.5 years after entering menopause, in no case coughing preceded menopause.

Chronic cough was clearly associated with an increase of menopausal symptoms and especially in regard to the subscales of the MRS II that mirror urogenital and somato-vegetative symptoms (Table 3, Fig. 1, Table 4). In addition, the results of the MRS and LCQ correlated with one another (Table 7). Furthermore, a significant prediction for chronic cough based on two somatic MRS domains and the total score could be shown (Table 8). These results contribute well to the biologically plausible theory that changes in the skin and mucous membranes can also show up in the respiratory tract, can change the sensitivity of the cough receptors and thus lead to increased cough sensitivity. The skin and mucous membranes represent an organ that can be influenced by hormonal changes. Decreasing estrogen level influence skin’s glycosaminoglycan content and promote tendency to create wrinkles, dryness, atrophy and poor wound healing [30, 36]. The collagen content of the skin is significantly reduced by 1–2% per year after menopause [37]. Some of these changes might also affect the respiratory system.While some research was published these possible connections have not yet been in the clinical focus of either pulmonologists or gynecologists. Furthermore, lung function is strongly influenced by the female cycle and menopause [20, 26].

Chronic refractory cough is a multifactorial symptom that can be the consequence of several pulmonary and extrapulmonary diseases including gastrooesophageal reflux, upper airway cough syndrome, obstructive sleep apnea, and medications such as ACE inhibitors [7, 8, 10, 38, 39]. However, in a number of patients the genesis of chronic cough remains unclear, despite extensive diagnostics.Then it is defined as chronic cough of unclear etiology or a chronic idiopathic cough (CIC). The clinical complaints of CIC, sometimes persisting for years, are a dry and excruciating cough, the urge to clear the throat and dysphagia [1, 2]. To define chronic cough solely on duration falls probably too short in clinical practice. While recent guidelines still use an 8 week basis definition, as it was done in the present study, inclusion criteria for novel treatments would e.g. demand chronic treatment refractory cough for more than a year.

While some patients suffer from daily cough others show remittent and relapsing course. Using a time based definition was probably appropriate for this analysis as it served the purpose of separating symptomatic women from the healthy [40].

According to recent literature, idiopathic cough occurs in up to 20–42% of cases of chronic cough and can be triggered by weak subclinical triggers such as gastric acid, thermal stimuli, or passive smoking [41]. In most cases, chronic idiopathic cough is difficult to treat as it does not respond to classic therapeutic options. Most patients treated in a cough clinic are not only female, but also postmenopausal [1, 15]. Due to the reaction to subclinical triggers, the clinical picture of chronic idiopathic cough is causally associated with cough hypersensitivity syndrome, which defines a disturbance in the sensory nerve function. The concept of hypersensitivity is described as a valid and clinically useful concept and can be seen as a neuropathy of the cough reflex [29, 35]. Accordingly, the cough receptors’ increased sensitivity and the changed central cough modulations lead to hyperreactivity of the cough reflex [30, 36, 42]. In postmenopausal women, the chronic cough stimulus may be due to the cough receptors’ increased sensitivity and/or a changed central cough modulation as well as changes in gastroesophageal reflux disease, laryngeal dysfunction or inflammation regulation [2, 12–15, 42]. However, according to the definition, no CIC can be assigned to the patients in this study, as they did not go through the necessary diagnostics.

The highly significant correlation of postmenopausal symptoms, especially the somato-vegetative subgroup and vaginal dryness and coughing, underline these assumptions and emphasize the need to further expand the complexity of the climacteric syndrome. MRS differences of 10–40% pre and post hormonal treatment on the score levels were described. Dinger and Heinemann suggested a statistical significant difference of at least 4 score points to be a reasonable goal for a direct head to head comparison of two medical treatments in menopause hormonal treatment. As not treatments but just coughing versus non coughing were compared in this study and subjects presented a difference of 3.63 (*p* < 0.001) between groups, this appears clinically relevant. Chronic (idiopathic) cough might be, in part, due to the lack of steroids in postmenopausal women and could be diagnosed and treated differently in the future. Further studies are needed to clarify the connections, the underlying pathomechanisms, and the effects of hormonal replacement therapy on the bronchopulmonary symptoms [35].

A limitation of this study it that it is a simple interview and questionnaire-based study with no additional information on individual findings such as mucus parameters, X-rays etc. On the other hand, a strength is that a reasonable and well-selected representative number of patients were involved. Also, the groups appeared to be well-balanced to baseline characteristics, the effects shown are statistically substantiated and the questionnaires that were used are validated and established tools to measure the aimed effects. At first glance a rate of 33% of rather healthy postmenopausal women that report coughing might seem high and was a surprising finding. On the other hand, existing cough was mainly defined by the time aspect of 8 weeks and majority of women reported rather mild symptoms. The LCQ values are lower in patients with COPD and chronically productive cough [43].

Therefore postmenopausal women might not be aware of their symptoms or connect them to their postmenopausal changes.

In addition, there is disagreement about the exact prevalence of chronic cough [44, 45].

## Conclusion

In conclusion, the results of our study demonstrate a clinically relevant, statistically significant relationship between climacteric symptoms and postmenopausal cough, along with a significantly reduced quality of life in women with postmenopausal chronic cough. These associations should raise awareness, regarding this previously unknown climacteric symptom serve as basis for further evaluation and should add another facet to the climacteric syndrome.

## Data Availability

The datasets used and/or analyzed during the current study are available from the corresponding author on reasonable request.

## Abbreviations

BMI: Body mass index
MRS: Menopause Rating Scale
LCQ: Leicester Cough Questionnaire
CIC: Chronic idiopathic cough
COPD: Chronic obstructive pulmonary disease
